# Urocortin Treatment Improves Acute Hemodynamic Instability and Reduces Myocardial Damage in Post-Cardiac Arrest Myocardial Dysfunction

**DOI:** 10.1371/journal.pone.0166324

**Published:** 2016-11-10

**Authors:** Chien-Hua Huang, Chih-Hung Wang, Min-Shan Tsai, Nai-Tan Hsu, Chih-Yen Chiang, Tzung-Dau Wang, Wei-Tien Chang, Huei-Wen Chen, Wen-Jone Chen

**Affiliations:** 1 Department of Emergency Medicine, National Taiwan University Medical College and Hospital, Taipei, Taiwan; 2 Division of Cardiology, Department of Internal Medicine, National Taiwan University Medical College and Hospital, Taipei, Taiwan; 3 Division of Cardiology, Department of Internal Medicine, Cardinal Tien Hospital Yunghe Branch, New Taipei City, Taiwan; 4 Graduate Institute of Toxicology, College of Medicine, National Taiwan University, Taipei, Taiwan; 5 Division of Cardiology, Department of Internal Medicine, Lotung Poh-Ai Hospital, Yilan County, Taiwan; Mount Sinai School of Medicine, UNITED STATES

## Abstract

**Aims:**

Hemodynamic instability occurs following cardiac arrest and is associated with high mortality during the post-cardiac period. Urocortin is a novel peptide and a member of the corticotrophin-releasing factor family. Urocortin has the potential to improve acute cardiac dysfunction, as well as to reduce the myocardial damage sustained after ischemia reperfusion injury. The effects of urocortin in post-cardiac arrest myocardial dysfunction remain unclear.

**Methods and Results:**

We developed a preclinical cardiac arrest model and investigated the effects of urocortin. After cardiac arrest induced by 6.5 min asphyxia, male Wistar rats were resuscitated and randomized to either the urocortin treatment group or the control group. Urocortin (10 μg/kg) was administrated intravenously upon onset of resuscitation in the experimental group. The rate of return of spontaneous circulation (ROSC) was similar between the urocortin group (76%) and the control group (72%) after resuscitation. The left ventricular systolic (dP/dt_40_) and diastolic (maximal negative dP/dt) functions, and cardiac output, were ameliorated within 4 h after ROSC in the urocortin-treated group compared to the control group (P<0.01). The neurological function of surviving animals was better at 6 h after ROSC in the urocortin-treated group (p = 0.023). The 72-h survival rate was greater in the urocortin-treated group compared to the control group (p = 0.044 by log-rank test). Cardiomyocyte apoptosis was lower in the urocortin-treated group (39.9±8.6 vs. 17.5±4.6% of TUNEL positive nuclei, P<0.05) with significantly increased Akt, ERK and STAT-3 activation and phosphorylation in the myocardium (P<0.05).

**Conclusions:**

Urocortin treatment can improve acute hemodynamic instability as well as reducing myocardial damage in post-cardiac arrest myocardial dysfunction.

## Introduction

Modern cardiopulmonary resuscitation (CPR) has been available for more than 50 years, although sudden cardiac arrest still has a high mortality rate. Many efforts have been made to improve the outcome for patients with sudden cardiac arrest. The 1-year survival rate is less than 10% even though the rate of return of spontaneous circulation (ROSC) is as high as 40–60% [1–3]. The damage of the cardiovascular system due to circulatory arrest and post-cardiac arrest syndrome leads to poor cardiac function and circulatory failure. Myocardial dysfunction and profound shock may exacerbate multiple organ failure, including the central nervous system, and cause mortality [4, 5]. Further neuroprotective intervention for post-cardiac arrest syndrome is infeasible during profound shock and hypotension. Managing post-cardiac arrest myocardial dysfunction can potentially improve cardiovascular failure and survival outcomes [6–8].

Urocortin is a novel peptide composed of 40 amino acids and is a member of the corticotrophin-releasing factor family. Type 2 receptors of corticotrophin-releasing factor have elevated levels in cardiomyocytes and vascular endothelial cells, while type 1 receptors are expressed at higher levels in the central nervous system. There are three different subtypes of urocortins, namely urocortin 1, 2 and 3 [9, 10]. Administration of urocortin-2 can improve the left ventricular systolic and diastolic function and decrease peripheral resistance in heart failure [11, 12]. In human studies of heart failure, patients who are treated with urocortin have increased cardiac outputs and significantly improved dyspnea symptoms [13, 14]. In addition to improving hemodynamic instability, urocortin can dilate the coronary artery and increase coronary blood flow. Ischemia reperfusion injury and the extent of myocardial infarction can be reduced by administering urocortin at the reperfusion stage [15].

In this study, we tested the hypothesis that urocortin treatment can improve acute hemodynamic instability and reduce myocardial damage in post-cardiac arrest myocardial dysfunction, in a preclinical setting. We administrated urocortin at the onset of CPR in an asphyxia-induced cardiac arrest model established by us [6]. Potential downstream signaling pathways were also investigated in addition to the hemodynamic functions and outcomes.

## Methods

The study design was approved by the Institution Review Board of the National Taiwan University College of Medicine and Public Health (permit number: 20120526). The investigation conforms to the Guide for the Care and Use of Laboratory Animals published by the US National Institutes of Health. All rats were housed in a rodent facility with a 12 h-light/ 12 h-dark cycle and ad libitum access to food and water prior to the experiment.

### Study design and setting

**Fig 1:**
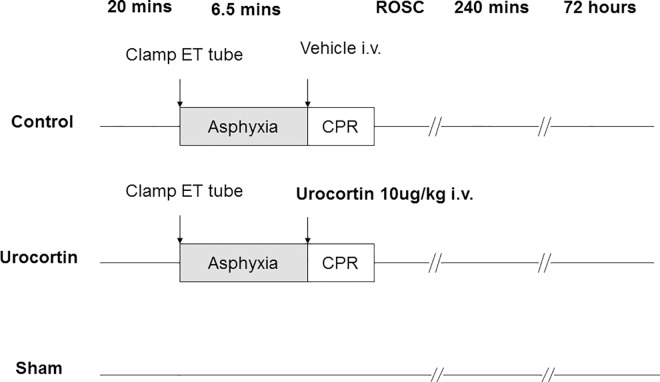
The study design and protocol for inducing cardiac arrest, resuscitation, urocortin treatment and monitoring.

A randomized animal study was implemented to investigate the effects of urocortin treatment. Urocortin-2 (10 μg/kg, Sigma-Aldrich, St. Louis, MO, USA) was intravenously administered at the onset of resuscitation after 6.5 min of asphyxia. The placebo control was treated with an equivalent volume of normal saline at the same time point (Fig 1). The most effective dose of urocortin for cardioprotective effects has been previously demonstrated [11, 16]. Animals in the sham group were surgically prepared without asphyxia-induced cardiac arrest and cardiopulmonary resuscitation.

Male Wistar rats (of 8 weeks old) were anesthetized with pentobarbital (30 mg/kg, i.p.) to minimize the possible suffering and distress caused to the animals during the procedures; prepared as previously described [6]. Anesthetic monitoring included testing of rear foot reflexes before any incision or intubation, and continuous observation of respiratory patterns, heart rates, and responsiveness to manipulations and rear foot reflexes throughout the procedure. No additional dose of anesthetic was used if resuscitated rats persistently showed no response or signs of suffering during the experiments. Tracheal intubation was performed with a PE 200 catheter (Angiocath, NJ, USA), and mechanical ventilation was then started. Arterial blood pressure, left ventricle (LV) pressure and central venous pressure were measured by means of cannulation through the right femoral artery, right carotid artery, and then advancing to the LV and right jugular vein. Pressure monitoring was performed with saline-filled PE 50 tubes. Fluids and drugs were administrated through the right jugular vein route. A PC-based data acquisition system (Powerlab, ADInstruments, CO, USA) was used to record pressures and needle-probe ECG monitoring data. LV systolic function was represented by dP/dt at 40 mmHg of LV pressure (dP/dt_40_) and diastolic function was represented by maximal negative dP/dt. A thermocouple microprobe was advanced to the proximal aorta through the left femoral artery. A Cardio-Max II system (Columbus Instruments, OH, USA) was used to measure the cardiac output with duplication by the thermodilution method.

### Asphyxia-induced cardiac arrest

Cardiac arrest was induced after preparation and stabilization of the animals. The ventilator was turned off and the endotracheal tube was clamped to induce cardiac arrest, which soon led to slowing of the heart rate and hypotension. An asystolic rhythm with undetectable blood pressure was then developed, and cardiac arrest was defined as mean femoral arterial pressure less than 10 mmHg. Adrenaline (0.005 mg/100 g) was injected through the venous line after 6.5 min of asphyxia. A pneumatically-driven mechanical chest compressor was then started at a rate of 300 beats/min. Coronary perfusion pressure was maintained at more than 20 mmHg during resuscitation. The respiratory rate was kept at 90 breaths/min and tidal volume was maintained at 0.8 mL/100 g with a fraction of inspired oxygen of 1.0 during chest compression. The fraction of inspired oxygen was adjusted to room air level (0.21), without any additional oxygen supplementation after ROSC. ROSC was usually achieved within 3 min, and animals were excluded if ROSC could not be obtained within 6 min.

### Outcome measures in survival and neurological function

Catheters were removed after 4 h of invasive monitoring. Wounds were surgically closed, and animals were extubated. Animals received an intra-peritoneal injection of 1 ml 0.9% saline at 1 h after extubation and were returned to their cages. Survival status of the different groups was monitored. To confirm the mortality status, which was the endpoint of the survival study for cardiac arrest and resuscitation, the health status of all rats was carefully monitored every 6 h. Mortality was confirmed by loss of spontaneous respiratory movements and loss of heart beat for more than 2 min. Recovery of neurological function was evaluated using the neurological functioning scores adapted from a grading system for rats [17] and recorded at 6, 24, 48, and 72 h after cardiac arrest. Evaluation was performed by two independent investigators who were blinded regarding which rats received urocortin treatment. If there were any discrepancies, a third investigator was invited to participate, and the scores chosen by the majority were accepted. No unexpected deaths were found during the careful monitoring in the study. Rats were euthanized with a lethal dose of pentobarbital sodium administered intraperitoneally, after completing the evaluation of survival and neurological status at 72 h after cardiac arrest.

### Harvesting of the heart and brain tissue

For studying myocardial damage and activation of signals related to the urocortin treatment, hearts were harvested at 2 h after cardiac arrest. Briefly, the chest was opened after injecting a lethal dose of phenobarbital (250 mg/kg) intra-peritoneally. Phenobarbital was used for euthanasia due to its rapid onset, with only minimal discomfort, and its feasible routes for intraperitoneal injection for rats [18]. The heart was retrieved from the aortic root and the LV was separated and excised from the right ventricle. All samples were immediately placed at -80°C and transferred to a liquid nitrogen tank for further investigation.

### Histological studies

To investigate the myocardial and cerebral damage, morphological and histological results of the hearts and brains were examined by hematoxylin and eosin staining. The apex, septum, and lateral wall of the LV were selected for evaluating myocardial injury. Terminal deoxynucleotidyl transferase dUTP nick-end labeling (TUNEL) staining was performed to detect DNA fragmentation with a colorimetric-terminal deoxynucleotidyl transferase enzyme (Calbiochem, San Diego, CA, USA), using methylene green as a nuclei counterstain in the myocardium [19]. Cells that were positive for TUNEL staining were counted in five randomly selected, independent microscopic fields at × 400 magnification in each heart specimen. In total, six myocardial specimens were counted per animal. The cornu amonis (CA1, CA2, CA3) in the hippocampus of each brain was selected for brain injury studies. To evaluate neuronal death, Nissl staining with cresyl violet (Sigma-Aldrich) was used [20]. Positive Nissl-stained neurons were counted in three independent, randomly selected microscopic fields in the cornu amonis of each brain specimen. In total, three hippocampal specimens were counted per animal. Histological examinations were performed by two independent pathologists who had been blinded to the groups. An independent assessment was carried out by a third investigator, and the majority opinion was chosen if there were any discrepancies.

### Western blot experiments to investigate the activation of downstream signaling pathways

Hearts were homogenized in 50 mmol/L Tris-HCl (pH 7.4), 1% nonylphenyl- polyethylene glycol, 0.25% sodium deoxycholate, 150 mmol/L NaCl, and 1 mmol/L ethylene glycol bis-2-aminoethyl ether-N,N,N,N-tetraacetic acid. Supernatants were aliquoted after centrifugation for immunoblot assays. Specific antibodies were used for recognizing the following proteins: total extracellular-signal regulated kinase (ERK) 42/44 or phosphorylated ERK 42/44 (Santa Cruz Biotechnology, Santa Cruz, CA, USA), total or phosphorylated Akt (Biosource International, Camarillo, CA, USA), total or phosphorylated STAT-3 at tyrosine 705 or serine 727 (Santa Cruz Biotechnology). Signals from immunoblots were scanned and quantified using the tool of ImagMaster TotalLab (Amersham Pharmacia Biotech, Uppsala, Sweden).

### Statistical analyses

Values of continuous variables were presented as mean ± standard deviation. The ANOVA test was applied for studying the significance of differences between groups for the continuous variables using the post-hoc test by Tukey’s HSD. We showed the survival of different groups by means of Kaplan-Meier curves and comparisons were made with the log-rank test. Generalized mixed linear models were used to compare the patterns and changes of hemodynamic parameters between urocortin-treated and control groups. Values of P<0.05 were considered statistically significant. Statistical analyses were performed using SPSS12.0 software (SPSS Inc., Chicago, USA).

## Results

### Baseline characteristics and resuscitation variables

Baseline characteristics and resuscitation variables, including cardiac arrest duration, end-tidal CO_2_ levels, coronary perfusion pressures and lactic acid levels were not significantly different between the control group and the urocortin group (Table 1). The ROSC rate was 72% in the control group and 76% in the urocortin group (p = 0.534). There were no significant differences of pH, pO_2_, pCO_2_ and HCO_3_ levels at ROSC or 2 h after ROSC between the two groups.

**Table 1:** Baseline characteristics and resuscitation variables.

	Control (n = 18)	Urocortin (n = 18)	P value
Body weight (g)	383.5 ± 50.1	373.1 ± 23.5	0.633
Body temperature (°C)	37.1 ± 0.4	36.9 ± 0.3	0.454
End-tidal CO_2_ at 15 sec (mmHg)	25.0 ± 8.2	27.8 ± 6.7	0.201
CPP^a^ at 90 sec (mmHg)	15.5 ± 5.1	15.1 ± 5.1	0.726
CPP at 180 sec (mmHg)	23.8 ± 5.2	23.6 ± 5.2	0.936
ROSC^b^ rate	72.0%	76.0%	0.534
Lactic acid at ROSC (mmol/L)	10.6±1.6	10.6±1.7	0.926
Cardiac arrest time (s)	193.9 ± 29.5	195.0± 33.1	0.881
Resuscitation time (s)	157.1 ±17.3	153.9 ± 13.7	0.923
Blood Gas Analysis at ROSC			
pH	6.96±0.10	6.92±0.07	0.269
PCO_2_ (mmHg)	70.5±10.3	72.3±7.7	0.654
PO_2_ (mmHg)	115.0±22.9	110.6±28.0	0.744
HCO_3_ (mEq/L)	15.9±3.4	15.4±3.2	0.676
Blood Gas Analysis at 2 h			
pH	7.29±0.11	7.30±0.03	0.304
PCO_2_ (mmHg)	44.5±5.3	46.0±6.0	0.495
PO_2_ (mmHg)	76.0±8.7	77.5±4.9	0.689
HCO_3_ (mEq/L)	25.3±3.7	25.5±2.6	0.904

^a^CPP: coronary perfusion pressure

^b^ROSC: return of spontaneous circulation

### Urocortin treatment improved the post-cardiac arrest cardiac function

**Fig 2:**
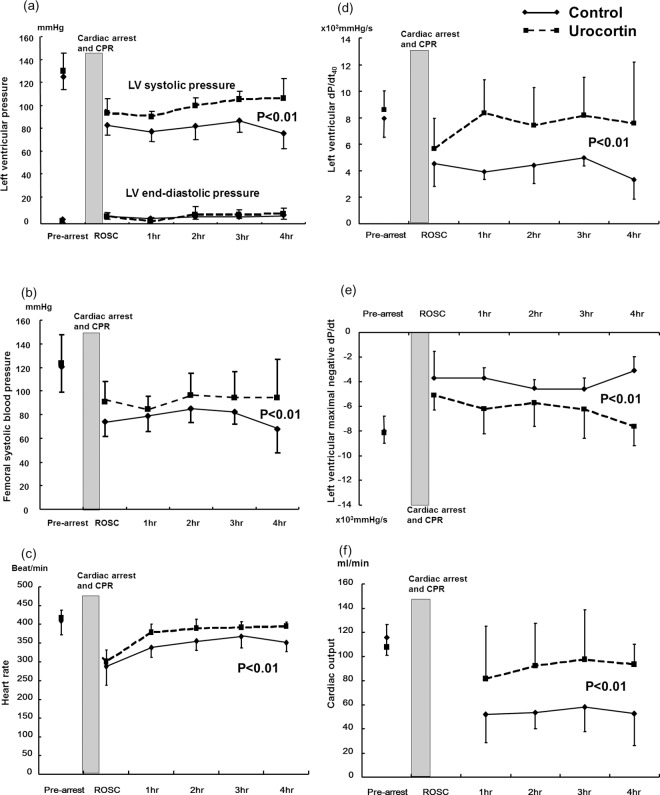
Hemodynamic parameters of control and urocortin groups from baseline to 4 h after cardiac arrest and resuscitation. (a) left ventricular systolic and diastolic pressure (b) femoral artery systolic blood pressure (c) heart rate (d) left ventricular systolic function represented by dP/dt_40_ (e) left ventricular diastolic function represented by maximal negative dP/dt (f) cardiac output (n = 10 in each group, P<0.01 between the control group and the urocortin group by generalized mixed linear analysis).

Hemodynamic parameters before the induction of cardiac arrest, and at ROSC, were not significantly different between the two groups (S1 Table). The left ventricular systolic pressure decreased after cardiac arrest and resuscitation in both groups (n = 10 in each group). The left ventricular systolic pressure and femoral artery systolic blood pressure were higher in the urocortin-treated group compared to the control group from 1 h to 4 h after ROSC (P<0.01, Fig 2A and 2B). The left ventricular systolic function, represented by dP/dt_40_, recovered better in the urocortin group (P<0.01, Fig 2D) and the diastolic function measured at maximal negative dP/dt was moderately better in the urocortin group (P<0.01, Fig 2E). The cardiac output decreased after cardiac arrest and CPR, and was undetectable just after ROSC. The recovery of cardiac output was better in the urocortin group at 1 h to 4 h post-cardiac arrest (P<0.01, Fig 2F).

### Survival study

**Fig 3:**
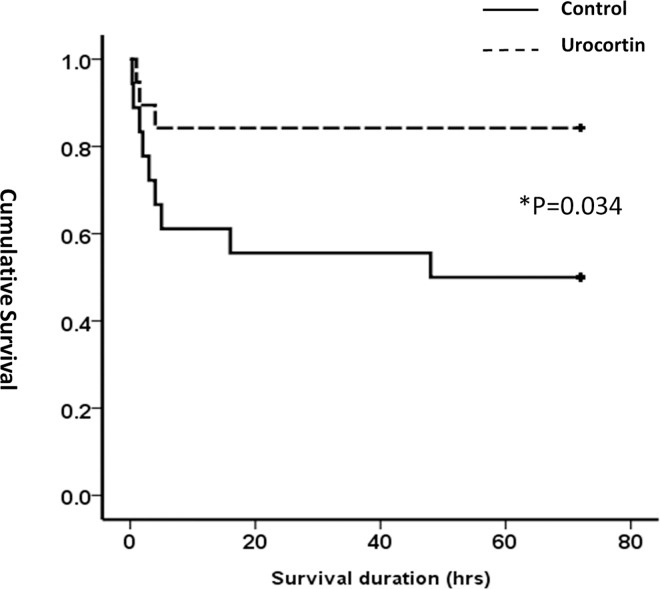
Kaplan-Meier survival curve of control and urocortin groups at 72 h after cardiac arrest and resuscitation. Better chances of survival at 72 h were noted with urocortin treatment (n = 18 in each group, *: P<0.05 between the two groups by log-rank test).

The survival statuses were followed up for 72 h, with a survival rate of 83.3% (15/18) in the urocortin group and 50.0% (9/18) in the control group (P = 0.034). Kaplan-Meier survival curve analysis showed a more rapid decline of survival rate in the control group in the early post-cardiac arrest period (Fig 3). The difference in survival rate persisted until 72 h after cardiac arrest and resuscitation (P = 0.044 by the log-rank test).

### Neurological function assessment

**Fig 4:**
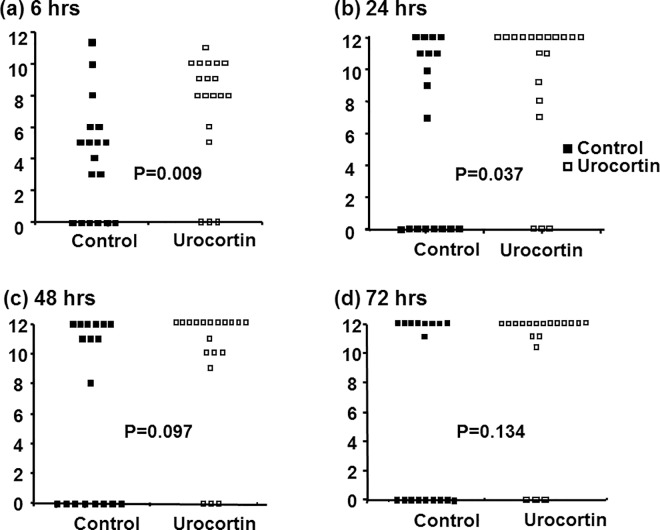
Neurological functioning scores of control and urocortin groups at 6, 24, 48 and 72 h after cardiac arrest and resuscitation. Neurological functions were better at 6 and 24 h in surviving subjects with urocortin treatment (n = 18 in each group, *: P<0.05 between the two groups).

The recovery of neurological function for surviving animals, assessed by the neurological functioning score system, was better in the urocortin group at 6 h and 24 h after cardiac arrest and resuscitation (P = 0.009 and 0.037 according to the Mann-Whitney test, respectively, Fig 4A and 4B). However, there were no significant differences between the groups in neurological scores at 48 h and 72 h after cardiac arrest in the surviving animals, although there was a higher mortality rate in the control group (Fig 4C and 4D).

### Histology studies for heart and brain damage

**Fig 5:**
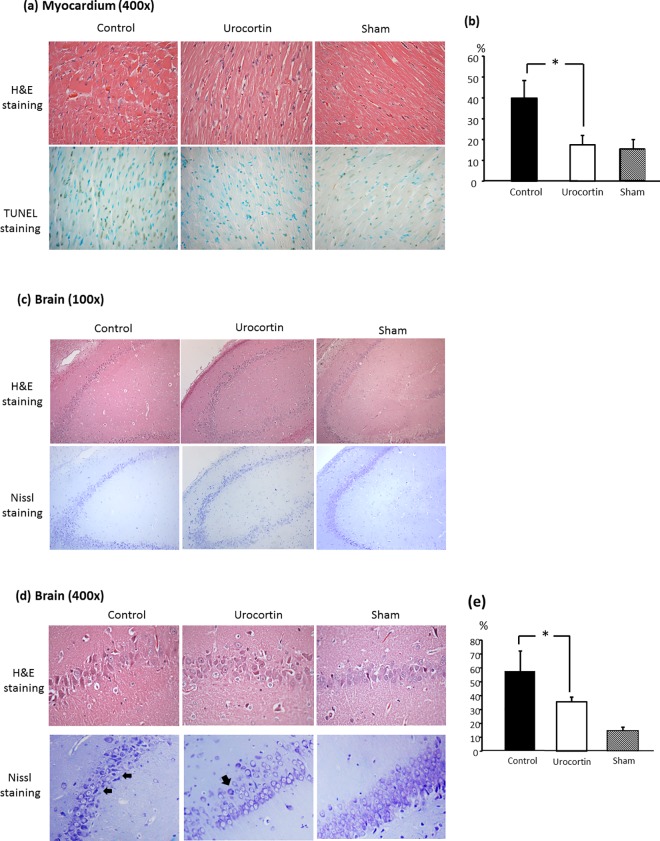
Histology studies of hearts and brains in control and urocortin-treated groups. (a) H&E staining and TUNEL staining for myocardium at 2 h after cardiac arrest. Fewer TUNEL-positive nuclei were noted in the urocortin group compared to the control group. (b) Quantification of TUNEL-positive nuclei in each group (n = 6 in each group, *: P<0.05) (c) & (d) H&E staining and Nissl staining in brains at 24 h after cardiac arrest with microscopic magnification of 100× and 400×, respectively. Fewer damaged positive-Nissl stained dark neurons (arrows) were noted in the urocortin group compared to the control group. (e) Quantification of Nissl-positive nuclei in each group (n = 6 in each group, *: P<0.05).

DNA fragmentation increased at 2 h after cardiac arrest and resuscitation with a greater number of TUNEL-positive nuclei in the myocardium. Urocortin treatment reduced cardiomyocyte apoptosis, with a lower number of nuclei with TUNEL-positive staining (39.9±8.6 vs. 17.5±4.6%, P<0.05, Fig 5A and 5B). In brain histological studies with Nissl staining, more severely damaged neurons with small-sized, darker and condensed cytoplasm were detected after cardiac arrest and resuscitation injury in Cornu amonis areas. However, there were fewer damaged neurons with positive Nissl staining found in the urocortin-treated group (57.6±14.4 vs. 35.6± 3.1%, P<0.05, Fig 5C–5E).

### Activation of downstream signaling pathways

**Fig 6:**
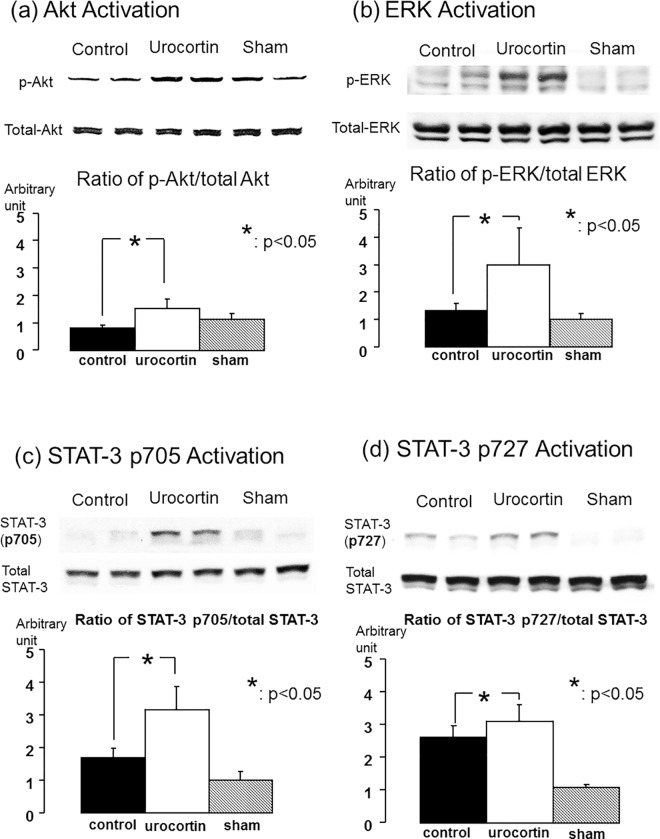
Protein expression by Western blotting images and quantification of signal transduction pathways in myocardium after cardiac arrest and resuscitation. (a) Akt (b) ERK42/44 in total lysates of myocardium at 2 h after cardiac arrest and resuscitation. Phosphorylation of Akt and ERK 42/44 was greater in the urocortin-treated group (*: P<0.05 for both). (c) & (d) Western blot showing STAT-3 phosphorylation with STAT-3 p-Tyr705 and STAT-3 p-Ser727 in the myocardium with and without urocortin treatment, respectively. After normalization to total amounts of STAT-3 protein, quantification of STAT-3 expression by Western blotting showed that more phosphorylated STAT-3 (Tyr 705) as well as STAT-3 (Ser 727) was expressed with urocortin treatment (n = 6 in each group, *: P < 0.05).

Activation and phosphorylation of Akt was significantly increased at 2 h after cardiac arrest and resuscitation in the urocortin group (n = 6 for each group P<0.05, Fig 6A). Levels of ERK 42/44 were moderately increased after cardiac arrest and resuscitation in the control group compared to the sham group. Administration of urocortin induced greater ERK phosphorylation compared to the control group (P<0.05, Fig 6B). STAT-3 was phosphorylated and activated at tyrosine 705 and serine 727 after cardiac arrest. Urocortin treatment induced further activation and phosphorylation of STAT-3 at both tyrosine 705 and serine 727 (P<0.05, Fig 6C and 6D).

## Discussion

Post-cardiac arrest myocardial dysfunction occurs after cardiac arrest and resuscitation and leads to mortality. Our study showed that urocortin treatment at the onset of CPR could not only improve the acute hemodynamic stabilization, but could also reduce myocardial damage and improve survival outcome during the post-cardiac arrest period. Urocortin treatment improved the left ventricular systolic and diastolic functions, and cardiac output was better preserved. Activation of downstream signal transduction pathways supports the reduced cardiomyocyte damage as a result of urocortin-related cardioprotective effects, and may contribute to improving the post-cardiac arrest myocardial dysfunction and survival outcome.

Post-cardiac arrest myocardial dysfunction and hemodynamic instability contributes to the poor survival rate following cardiac arrest [21]. Although some studies have shown that myocardial dysfunction is reversible at 48 to 72 h after ROSC, the low cardiac output and poor peripheral circulation in the early post-resuscitation period exacerbates the global ischemia reperfusion injuries in cardiac arrest [22]. Supporting the circulatory system during CPR and in the post-resuscitation period can improve the critical hemodynamic status and survival outcome [7, 23]. Urocortin has been demonstrated to be effective in treating acute decompensated heart failure due to its strong inotropic and lusitropic effects on the LV, with decreased systemic arterial vascular resistance [11, 13, 24]. Patients with post-cardiac arrest myocardial dysfunction present with acute cardiac failure due to severe global ischemia reperfusion injuries, which differs from acute cardiac failure due to decompensation from chronic heart failure [25, 26]. The effect of urocortin on post-cardiac arrest myocardial dysfunction has never been previously reported. In our study, we found that urocortin can exert positive inotropic and lusitropic effects in the cardiac arrest model. Urocortin was able to induce early improvement and stabilization of circulatory failure in the post-cardiac arrest period, thus improving survival outcome in experimental animals, as shown in this study.

Urocortin exerts its positive inotropic and lusitropic effects via different mechanisms compared to dobutamine. Pre-treatment of the beta-receptor blocker esmolol does not block the beneficial urocortin-induced hemodynamic effects or decrease the heart rate [11]. The acute effect on improving cardiac performance can arise from an increase in cAMP-dependent PKA activity, phosphorylation of phospholamban, and reduced inhibition of sarcoplasmic reticulum calcium–ATPase upon activation of corticotropin-releasing factor receptor-2 by urocortin [27, 28]. Inotropic agents are often needed to maintain an adequate cardiac output and organ perfusion for hemodynamic unstable patients during the post-cardiac arrest period. Pivotal therapy, such as targeted temperature treatment, could be infeasible in conditions of hemodynamic instability with significant low blood pressure [29]. However, profound shock persists even under high doses of catecholamine in the post-cardiac arrest period [30]. Side effects of high doses of catecholamine then occur, which cause further deterioration of the organ perfusion. This suggests a role for urocortin as an early parenteral therapy in managing post-cardiac arrest myocardial dysfunction and circulatory failure. Alternative therapies from the traditional catecholamine-based inotropic agents introduce urocortin as one of the components in the combination “cocktail” treatment for cardiac arrest and resuscitation [27].

Neurological injuries occur after cardiac arrest and determine long-term functional outcome. Although it has been reported that urocortin is beneficial for neuron protection [31], the ability of urocortin to cross the blood-brain-barrier has been shown to be very low in adult mice [32]. The neurological benefit was only observed in the first 24 h post-resuscitation in this study. It seems that the observed difference is due to the poor neurological outcome of the untreated animals, which were very sick and died during the day 1. Therefore, a stable neurological benefit could be questionable in this model. The reduced neuron damage and improved recovery of neurological function after cardiac arrest and resuscitation as a result of urocortin could be due to a better hemodynamic status and brain perfusion, rather than a direct urocortin-induced neuron protection effect.

Myocardial ischemia reperfusion injuries occur after cardiac arrest and are potential therapeutic targets for cardioprotection. Apoptosis can be ameliorated by cardioprotective treatment against ischemia reperfusion injuries [33]. In our study, we found that urocortin treatment was able to reduce myocardial apoptosis after cardiac arrest and resuscitation in addition to having beneficial hemodynamic effects. It has been reported that urocortin treatment is associated with downregulation of apoptosis and improvement of myocardial injury in an *ex vivo* isolated cardiac ischemia reperfusion injury model [34]. These results support our findings of reduced apoptosis in the myocardium with urocortin treatment after cardiac arrest and resuscitation. The mechanisms of decreased apoptosis and cardioprotective effects arise from several different signaling pathways due to urocortin treatment. The increase in cAMP concentration activates protein kinase A after urocortin treatment and also facilitates ERK phosphorylation [15, 35, 36]. This pathway activates the pro-survival Bcl-2 family and prevents the mitochondria permeability transition opening and apoptosis [37]. Another pathway activated upon urocortin treatment is the phosphoinositide 3-kinase (PI3K) pathway through the recruitment of the G-protein in corticotrophin-releasing factor receptor type 2 (CRFR2). As a result, the downstream protein kinase B (Akt) is phosphorylated and activated [28, 38]. Activation of Akt leads to the sequestration of the pro-apoptotic protein BAD in the cytosol, which reduces the levels of free BAX and inhibits the further activation of apoptotic pathways [37]. Interaction of Akt and STAT-3 pathways can reduce myocardial damage and can play important roles in improving mitochondria functions in post-cardiac arrest myocardial dysfunction [39, 40]. Activation and serine phosphorylation of the STAT-3 pathway has been found to be associated with preservation of mitochondrial integrity and a reduction in post-cardiac arrest myocardial dysfunction [40]. Activation of STAT-3 has not been extensively investigated, but urocortin treatment has been found to induce STAT-3 activation [41]. In this study, we found that urocortin treatment can activate key pro-survival signal transducers including Akt, ERK and STAT-3 after cardiac arrest and resuscitation. These findings imply that activation of cardioprotective signaling pathways by urocortin treatment may support its protective effects against post-cardiac arrest myocardial injuries.

Our study does, however, have some limitations. First of all, cardiac arrest was induced by asphyxia and occurred as a non-ventricular fibrillation model. It is unknown whether the beneficial effects are the same in the ventricular fibrillation cardiac arrest model, and thus requires further investigation. Secondly, our study was performed on healthy animals without any known existing diseases. The impact of underlying diseases on the responses to urocortin treatment is not clear. More studies on the diseased model are thus needed to clarify this issue. Treatment for post-cardiac arrest syndrome in the model follows the current guidelines, apart from hypothermia treatment. For initially elucidating the net effect of urocortin treatment on post-cardiac arrest myocardial dysfunction, hypothermia treatment was not applied in the study. Further studies are needed before translating the urocortin cardioprotective effects to a clinical application.

In conclusion, myocardial dysfunction occurred after cardiac arrest and resuscitation in the cardiac arrest model. Urocortin treatment at the onset of CPR can improve acute hemodynamic instability with better preserved left ventricular systolic and diastolic functions. Myocardial damage was reduced as a result of urocortin, with less apoptosis; urocortin-related pro-survival kinases were activated, including Akt and ERK. These findings support the potential clinical applications of urocortin in ameliorating the post-cardiac arrest myocardial dysfunction and survival outcome following cardiac arrest.

## Supporting Information

S1 Table Hemodynamic data and cardiac function before inducing cardiac arrest and after resuscitation were not significantly different between the groups. (DOCX)
