# Supplementary material for: Urocortin Treatment Improves Acute Hemodynamic Instability and Reduces Myocardial Damage in Post-Cardiac Arrest Myocardial Dysfunction
Source: PLoS One. 2016 Nov 10;11(11):e0166324. doi: 10.1371/journal.pone.0166324 (PMC5104489; doi:10.1371/journal.pone.0166324)
Supplement: S1 Table — (DOCX) [file pone.0166324.s001.docx]

Table S1: Hemodynamic data and cardiac function before inducing cardiac arrest and after resuscitation were not significantly different between the groups

| Time point | **Pre-arrest** | **ROSC** | **1hr** | **2hr** | **3hr** | **4hr** |
| --- | --- | --- | --- | --- | --- | --- |
| **Left ventricular systolic pressure (mmHg)** | | | | | | |
| Control | 123.2± 10.9 | 81.3±8.7 | 75.8±8.5^a^ | 81.1±11.9^a^ | 85.5±10.4^a^ | 74.3±13.4^a^ |
| Urocortin | 128.5±15.7 | 91.6±13.2 | 88.9±4.3 | 97.9±7.7 | 104.2±7.1 | 104.2±17.6 |
| **Left ventricular end-diastolic pressure (mmHg)** | | | | | | |
| Control | 3.4±2.4 | 6.0±2.1 | 4.5±2.3 | 5.7±2.3 | 6.3±2.0 | 7.4±4.0 |
| Urocortin | 2.4±1.6 | 5.8±4.2 | 1.8±3.4 | 7.9±6.9 | 7.6±4.5 | 8.1±5.4 |
| **Femoral systolic blood pressure (mmHg)** | | | | | | |
| Control | 120.4±21.5 | 73.9±12.7 | 78.7±13.1 | 84.9±11.6 | 81.8±10.5 | 67.4±20.1 |
| Urocortin | 123.3±24.0 | 90.5±17.3 | 84.5±10.5 | 96.2±18.9 | 93.9±22.2 | 94.0±32.6 |
| **Heart rate (beat/min)** | | | | | | |
| Control | 410.1±38.4 | 288.7±50.9 | 338.2±25.5^a^ | 355.8±25.4^a^ | 367.5±30.5 | 352.2±25.3 |
| Urocortin | 416.2±21.9 | 300.7±31.5 | 379.1±20.7 | 389.7±24.6 | 392.7±14.8 | 394.7±11.0 |
| **Left ventricular dP/dt_40_ (mmHg/s)** | | | | | | |
| Control | 7948.8±1383.2 | 4539.5±1728.0 | 3944.2±588.6^a^ | 4419.5±1375.5^a^ | 4999.5±640.7^a^ | 3340.1±1484.0^a^ |
| Urocortin | 8612.5±1423.0 | 5655.4±2308.6 | 8326.5±2551.2 | 7406.1±2859.0 | 8182.7±2881.8 | 7590.8±4584.0 |
| **Left ventricular maximal negative dP/dt (mmHg/s)** | | | | | | |
| Control | 8004.3±1518.5 | 3703.3±2171.8 | 3709.5±824.9^a^ | 4488.7±648.4 | 4579.9±886.2 | 3072.9±1103.1^a^ |
| Urocortin | 8133.9±883.9 | 5098.7±1207.5 | 6192.0±2028.1 | 5704.2±1928.6 | 6220.8±2365.1 | 7638.1±1533.6 |
| **Cardiac output (ml/min)** | | | | | | |
| Control | 115.8±14.8 | NA^b^ | 52.0±23.4 | 53.6±13.7^a^ | 58.2±20.7 | 52.6±26.4^a^ |
| Urocortin | 107.7±18.5 | NA^b^ | 81.4±43.9 | 92.3±35.4 | 97.4±41.2 | 93.5±16.6 |

a: There was statistically significantly difference (P< 0.05) between control and urocortin group at the same time point by t-test.

b: NA: Cardiac output decreased after cardiac arrest and CPR and was undetectable just after ROSC.
